# Characterization and Function of MicroRNA^∗^s in Plants

**DOI:** 10.3389/fpls.2017.02200

**Published:** 2017-12-22

**Authors:** Wei-wei Liu, Jun Meng, Jun Cui, Yu-shi Luan

**Affiliations:** ^1^School of Life Sciences and Biotechnology, Dalian University of Technology, Dalian, China; ^2^School of Computer Science and Technology, Dalian University of Technology, Dalian, China

**Keywords:** miRNA, miRNA^∗^, passenger strand, ARGONAUTE protein, stress response

## Abstract

MicroRNAs, a group of non-coding RNA molecules, play essential roles in a wide range of cellular processes in different molecules, cells, and organisms. In plants, microRNAs are a class of 20- to 24-nucleotides endogenous small RNAs that repress gene expression. The microRNA guide strand (miRNA) and its complementary strand (miRNA^∗^) both originate from the miRNA/miRNA^∗^ duplex. Generally, the guide strands act as post-transcriptional regulators that suppress gene expression by cleaving their target mRNA transcripts, whereas the complementary strands were thought to be degraded as ‘passenger strands.’ However, the complementary strand has been confirmed to possess significant biological functionality in recent reports. In this review, we summarized the binding characteristics of the miRNA^∗^ strands with ARGONAUTE proteins, their tissue-specific accumulations and their biological functions, illustrating the essential roles of miRNA^∗^s in biological processes and therefore providing directions for further exploration.

## Introduction

MicroRNAs are a group of non-coding RNAs that were first discovered as temporal regulators of larval differentiation in the nematode *Caenorhabditis elegans* (Lee et al., 1993); microRNAs play important roles in the control of diverse cellular pathways and participate in most biological processes in both plants and animals (Bartel and Bartel, 2003; Stefani and Slack, 2008). *In vivo*, the maturation of microRNAs requires a series of complex processes. First, endogenous genes are transcribed by RNA polymerase II (Pol II) into long primary microRNAs (pri-miRNAs) ranging from hundreds to thousands of nucleotides in length; pri-miRNAs are polyadenylated, single-stranded RNA molecules that fold into hairpin-like structures (Cai et al., 2004; Lee et al., 2004). The pri-miRNAs are then processed by the endonuclease RNase III. The species of RNase III and the mechanisms of further microRNA processing differ significantly between animals and plants (Du and Zamore, 2005). Unlike animals, plants lack Drosha homologs. Thus, after the formation of pri-miRNAs, the RNase III enzyme DICER-LIKE 1 (DCL1) regulates both the first step, which in animals involves cuts made by Drosha, and the second step, which in animals is reprocessing by Dicer, with the aid of HYPONASTIC LEAVES 1 (HYL1) and Serrate (SE); this process produces a miRNA/miRNA^∗^ duplex in the nucleus (Chen, 2009; Voinnet, 2009). The mature microRNA duplex consists of the active miRNA strand, called the guide strand, and the complementary miRNA^∗^ strand, called the passenger strand. Recently, the liberated strands have also been defined as miRNA-3ps and miRNA-5ps, according to the 5′ and 3′ arms of the hairpin precursor, after renaming by the miRBase registry (Kozomara and Griffiths-Jones, 2014; Yaish et al., 2015). The imperfect pairing of the miRNA/miRNA^∗^ duplex results in variable stability at the 5′ ends of the two strands. Once liberated from the duplex, the guide strands with lower thermodynamic stability at the 5′ end and high abundance are commonly loaded into specific ARGONAUTE (AGO)-associated RNA-induced silencing complexes (RISCs) and guide the RISCs to their targets. Originally, the passenger strands were thought to be degraded, since the accumulation of most passenger strands is much lower than that of the guide strands, and thus miRNA^∗^s were presumed to be mere by-products of the miRNA biogenesis pathway (Khvorova et al., 2003; Schwarz et al., 2003). However, an increasing number of reports have demonstrated that miRNA^∗^s may also act as important regulatory factors in organisms, and a large number of miRNA^∗^s have been confirmed to possess DCL1-dependency as similar to the miRNAs in Arabidopsis and rice (Meng et al., 2011).

## The Combination Preferences of Specific Ago Proteins

In plants, miRNAs are involved in RISCs and induce the repression of gene expression in either a transcriptional or a post-transcriptional manner. One difference between animal and plant miRNAs is that the latter bind to regulatory targets with highly complementary recognition sites, a general characteristic of miRNA-guided cleavage actions (Jones-Rhoades et al., 2006; Voinnet, 2009). Because of relaxed selection pressure, miRNA^∗^s are generally less conserved than miRNAs and more often polymorphic in both animals and plants (Guo and Lu, 2010; Smith et al., 2015). In recent years, several reports have also indicated that miRNA^∗^s take part in gene regulation via incorporation into AGO-associated RISCs in both animals and plants (Okamura et al., 2008; Ghildiyal et al., 2010; Zhou et al., 2010; Zhang et al., 2011).

**FIGURE 1:**
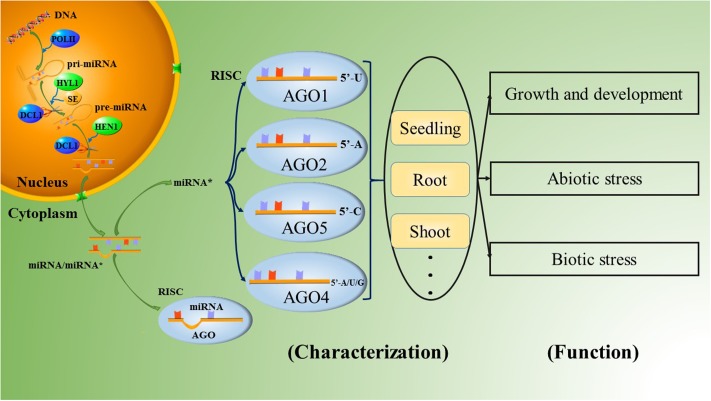
The biological process of the miRNA^∗^ in plants.

The AGO family in plants has undergone extensive diversification, giving rise to plant-specific AGOs (Poulsen et al., 2013), including 10 AGO proteins in Arabidopsis. Among them, AGO1 has been demonstrated to be associated with most miRNAs, forming RISCs to cleave the corresponding targets (Qi et al., 2005, 2006; Zhang et al., 2011). Furthermore, AGO1 has a preference for sequences with a uridine nucleotide at the 5′ terminus (Qi et al., 2006). Some of the other AGO proteins also have slicer activity. Takeda et al. (2008) analyzed the roles of AGO2 and AGO5 in *Arabidopsis thaliana*. AGO2- and AGO5-associated small RNAs were initially found to have obvious preferences for the nucleotides adenine and cytosine at their respective 5′ ends by 5′ labeling of the immunoprecipitated small RNAs. The two most abundantly cloned small RNAs were miR163-LL and miR390 in the AGO2 library and miR163-UL and miR390^∗^ in the AGO5 library; these molecules could form the small RNA duplexes miR163-LL/miR163-UL and miR390/miR390^∗^. (Note that the small RNAs derived from the lower left and the upper left of mature miR163 in pre-miR163 were named miR163-LL and miR163-UL, respectively.) Furthermore, if the 5′ nucleotides of miR163-LL and miR163-UL are exchanged, the strand of the miR163-LL/miR163-UL duplex that is preferentially incorporated into either AGO2 or AGO5 is also exchanged. This result indicates that the 5′ nucleotide plays an important role in guide strand selection in both AGO2 and AGO5 to form specific AGO-small RNA complexes in Arabidopsis. This article also provides evidence that the ‘passenger strand,’ which was previously thought to have no function, forms a RISC complex by combining with an AGO protein, functioning as an additional guide strand, as shown by miR390^∗^ in AGO5 (Takeda et al., 2008). The other typical species of AGO2-bound small RNA is miR393b^∗^, which possesses an adenine at the 5′ end and mediates disease resistance by targeting a Golgi-localized SNARE gene, *MEMB12* (Zhang et al., 2011; Zhang Z. et al., 2016). Overall, *Arabidopsis thaliana* AGO1 (AtAGO1) prefers sequences with a uridine nucleotide at the 5′ end, such as miR170a^∗^, miR171a^∗^ and miR173^∗^; AtAGO2 favors those with a 5′ adenine, such as miR393b^∗^ and miR837-5p^∗^; AtAGO5 has a bias toward miRNA^∗^s with a 5′ cytosine, such as miR158a^∗^ and miR390a^∗^; and AtAGO4 accepts variable 5′-terminal nucleotides, especially adenine, uridine or guanine (**Figure 1**; Mi et al., 2008; Takeda et al., 2008; Meng et al., 2011; Wang et al., 2011; Zhang et al., 2011, 2015; Manavella et al., 2012, 2013). However, the 5′-terminal nucleotide is not the only determinant of miRNA^∗^ combination with AGO. For example, the abundant miRNA^∗^s of miR396a and miR396b in Arabidopsis are designated miR396a-3p and miR396b-3p, respectively. Both of these molecules accumulated on AGO2 with a 5′-terminal guanine instead of a 5′-terminal adenine. In addition, miR396a-3p was also found to accumulate on AGO1 and AGO4 (Jeong et al., 2013).

## Characteristics of miRna^∗^ Accumulation

In the past, it was generally believed that the accumulation of miRNA^∗^s in organisms was much lower than that of miRNAs. Nevertheless, several studies have demonstrated that many miRNA^∗^s have abundances similar to or higher than their corresponding miRNAs in specific biological processes, in specific tissues and at specific times. For instance, miR156a^∗^, miR164b^∗^, and miR535^∗^ of *Betula luminifera*; miR82^∗^ of *Morus notabilis* in leaf tissue; miR166^∗^, miR159^∗^, and miR171^∗^ of *Orchis italica*; miR171c^∗^, miR369a^∗^, and miR2612a^∗^/b^∗^ of *Medicago truncatula*; and miR169h^∗^, miR408^∗^, and miR529a^∗^ of rice were all more abundant than their corresponding miRNAs (Devers et al., 2011; Peng et al., 2011, 2013; Aceto et al., 2014; Jia et al., 2014; Yan et al., 2014; Zhang J. et al., 2016). However, these miRNA^∗^s did not necessarily maintain this trend at all times. For example, rice miR1425^∗^ accumulation was lower than that of miR1425 at the later grain filling stage, indicating that it played an important physiological role in rice grain filling (Peng et al., 2013). Some miRNA^∗^s also show similar abundance to their partners, such as *Morus notabilis* miR166^∗^, miR76a-2^∗^, and miR82^∗^ in male flowers; miR172^∗^ and miR399c^∗^/j^∗^/k^∗^ of *Medicago truncatula*; and miR162c^∗^, miR162b^∗^, miR157a^∗^ and others of *Brassica oleracea* (Devers et al., 2011; Lukasik et al., 2013; Jia et al., 2014).

The tissue specificity of miRNA^∗^s is reflected in their different patterns of abundance. Some preferentially accumulate in specific tissues. In rice, miR169g^∗^, miR169p^∗^, and miR396c^∗^ were dominantly distributed in seedlings; miR396a^∗^, miR396b^∗^, and miR2121a^∗^ were accumulated in the roots; miR5159^∗^ and miR1423b^∗^ were highly accumulated in shoots; miR1428f^∗^ was highly expressed in the inflorescences; and miR166e^∗^, miR1433^∗^, and miR5533^∗^ were expressed in the panicles, indicating their potential activities in rice development (Shao et al., 2013; Hu et al., 2014). Similarly, in Arabidopsis, miR396a^∗^ was nearly undetectable in roots but was detected in flowers, and miR172b^∗^ was detected in leaves; meanwhile, miR2111a^∗^ and miR2111b^∗^ accumulated in seedlings (Meng et al., 2012; Jeong et al., 2013). Manavella et al. (2013) have confirmed miR171a^∗^ as important for normal development in Arabidopsis; this miRNA^∗^ triggers silencing of *SU(VAR)3-9 HOMOLOG8* in specific tissues such as the shoot apical meristem, the tops of anther filaments, the bases of young leaves, and the stomata. Interestingly, in *Medicago truncatula*, miR169d^∗^/l^∗^/m^∗^/e.2^∗^ target *MtBcp1*, which is localized in the plasma membrane and shapes the plasma membrane to the perihyphal membrane; these species were more abundant in mycorrhizal roots than in non-mycorrhizal roots, indicating that they played a role in mycorrhizal symbiosis by causing the roots to become more conducive to the absorption of nutrition (Pumplin and Harrison, 2009; Devers et al., 2011). Furthermore, the formation of some miRNA^∗^s is related to the growth phases of plants, such as miR172^∗^ and miR390^∗^ in cotton. These miRNA^∗^s were up-regulated in seedlings but down-regulated in other growth stages, while cotton miR171^∗^, miR2949^∗^ and others were regulated in the opposite manner (Xie and Zhang, 2015). Therefore, miRNA^∗^s can be specifically expressed in different tissues to maintain the steady state of the organism.

## Responses to Stress

With further research into miRNA^∗^s, more of them have been confirmed to respond to many stresses. As an example, miR399b-5p in barley, which was defined as the passenger strand, showed a much higher increase in expression under P deficiency than that of the guide strand (Hackenberg et al., 2013). An inverse result was found for miR169g^∗^ and miR172b^∗^ in tomato leaves; they were down-regulated under Pi deficient conditions compared with Pi sufficiency (Gu et al., 2010). Recent studies have indicated that nitrate significantly affects the expression of several miRNA^∗^s, such as miR169i^∗^/j^∗^/k^∗^ and miR528a^∗^/b^∗^ in maize roots (Trevisan et al., 2012). MiR169^∗^ possesses a large number of targets in sugarcane. Specifically, Elongation Factor 1-alpha (EF 1-α), which was encoded by the majority of miR169^∗^ targets, has been identified in response to abiotic stress such as water depletion. Thus, many miRNA^∗^s participate in regulating more than one stress response. In addition, miRNA^∗^s also respond to other stresses, such as gamma irradiation and high salt (**Table 1**).

**Table 1:** Biological function of miRNA^∗^s in plants.

Species	miRNA^∗^s in abiotic stress//biotic stress//development	Cited Reference
Arabidopsis	miR169^∗^, miR172b^∗^, miR778^∗^, miR2111^∗^, miR399^∗^, miR396b^∗^//miR393^∗^, miR825^∗^//miR171a^∗^	(Pant et al., 2008; Hsieh et al., 2009; Barciszewska-Pacak et al., 2015; Kim et al., 2016; Du et al., 2017)//(Zhang et al., 2011; Niu et al., 2016)// (Manavella et al., 2013)
Rice	miR1320^∗^, miR1425^∗^, miR160a^∗^//miR160a^∗^-f^∗^, miR166a^∗^-e^∗^/g^∗^-l^∗^/n^∗^, miR167a^∗^/c^∗^-e^∗^/h^∗^/i^∗^, miR171c^∗^-f^∗^/i^∗^, miR396a^∗^-c^∗^/e^∗^/f^∗^, miR1318^∗^, miR1425^∗^, miR159a^∗^, miR168^∗^, miR172d^∗^, miR390^∗^, miR444b.2^∗^, miR528^∗^//	(Hu et al., 2014)//(Du et al., 2011)//
Rapeseed	miR398a^∗^, miR399a^∗^, miR399c^∗^, miR399d^∗^, miR399f^∗^, miR778^∗^, miR2111a^∗^, miR2111b^∗^//	(Pant et al., 2009)//
Barley	miR399b^∗^, miR399c^∗^, miR528b^∗^//	(Hackenberg et al., 2013; Liu and Sun, 2017)//
Cotton	//miR172^∗^, miR390^∗^, miR171^∗^, miR2949^∗^, miR3954^∗^, miR164^∗^	//(Xie and Zhang, 2015)
Birch	//miR396c^∗^//	//(Zhang J. et al., 2016)//
Tomato	miR169g^∗^, miR172b^∗^//	(Gu et al., 2010)//
Maize	miR533a^∗^, miR169i^∗^/j^∗^/k^∗^, miR528a^∗^/b^∗^//	(Trevisan et al., 2012; Casati, 2013)//
Sugarcane	miR169^∗^, miR396^∗^, miR399^∗^//	(Thiebaut et al., 2014)//
Chinese cabbage	miR1885b.3^∗^, miR1885b.2^∗^//	(Yu et al., 2012)//
Switchgrass	miR169^∗^//	(Hivrale et al., 2016)//
Populus	miR396e^∗^//	(Chen et al., 2015; Ren et al., 2015)//
Barrel medic	//miR169d^∗^/l^∗^/m^∗^/e.2^∗^	//(Devers et al., 2011)
Soybean	//miR1511^∗^	//(Luo et al., 2012)

The roles of miRNA^∗^s in the regulation of biological stresses (**Figure 1**) have mainly been studied in Arabidopsis and rice to date. Arabidopsis miR825^∗^, a 22-nt small RNA that can potentially initiate the production of secondary siRNAs (Zhai et al., 2011; Li et al., 2012), targets toll-interleukin-like receptor (TIR)-nucleotide binding site (NBS) and leucine-rich repeat (LRR)-type resistance (*R*) genes to enhance resistance to *Pseudomonas syringae* pv. *tomato* (*Pst*) DC3000 infection in a manner strictly dependent on *Bacillus cereus* AR156 pretreatment (Niu et al., 2016). In *Rice stripe virus* (RSV)-infected rice plants, many miRNA^∗^s were also found to accumulate at higher levels than in normal plants; these miRNA^∗^s include the miR160^∗^, miR166^∗^, and miR396^∗^ families (Seo et al., 2013).

## Co-Regulation

miRNA^∗^s are more divergent than miRNAs, but there is no doubt about their importance to biological functions (Smith et al., 2015; Jain and Das, 2016). miRNA^∗^s cannot only function independently in biological processes, moreover, some miRNA^∗^s and their corresponding miRNAs act together to regulate intracellular activity (Hsieh et al., 2009; Zhang et al., 2011; Hu et al., 2014; Niu et al., 2016; Liu and Sun, 2017). Zhang et al. (2011) explored the function of miR393/miR393^∗^ in *Arabidopsis*, showing that miR393 is loaded into AGO1 and mediates pathogen-associated molecular pattern (PAMP)-triggered immunity (PTI) by negatively regulating auxin signaling pathways, whereas miR393^∗^ is loaded into AGO2 and mainly regulates plant effector-triggered immunity (ETI) responses by suppressing *MEMB12* and promoting the exocytosis of antimicrobial PR proteins; thus, both strands contribute to antibacterial responses in a synergistic manner (Navarro et al., 2006). Analogously, miR825 and miR825^∗^ were significantly down-regulated in *Pst* DC3000-infected *Arabidopsis* plants after *Bacillus cereus* AR156 pretreatment, and both of them were confirmed suppressing genes involved in bacterial pathogen defense (Niu et al., 2016). In addition, other experimental results have shown that miRNA and the corresponding miRNA^∗^ cannot only regulate a common biological pathway but also cooperate to cleave a single target gene. Luo et al. (2012) found that both miR1511 and miR1511^∗^ in soybean cleaved the target gene *GmRPL4a*, which belongs to the 60S ribosomal protein L4 family and shows a greater than 80% similarity to the RPL4A and RPL4D proteins in *Arabidopsis*. Therefore, they suggested that miR1511/1511^∗^ may function in regulating the development of soybean leaves (Luo et al., 2012).

Furthermore, because of the high complementarity between miRNA and miRNA^∗^ sequences, certain miRNA^∗^s could potentially act as anti-miRNAs to inhibit the binding of their homologous miRNAs to the target transcripts, subsequently reducing the activities of those miRNAs (Ma et al., 2015). Meanwhile, the accumulation of the miRNA^∗^ might regulate the mature miRNA or the precursor itself (German et al., 2008; Li et al., 2010; Meng et al., 2010). In addition, base mutations of one strand in the duplex may result in binding to a different AGO protein (Zhang and Zhang, 2012; Zhang et al., 2015). However, little research exists on the self-regulation of miRNA^∗^, and thus, this aspect needs to be further explored to deepen our understanding.

## Conclusion

miRNA^∗^, the passenger strand of the miRNA/miRNA^∗^ duplex, was generally thought to be degraded after the formation of mature miRNAs. However, all studies of miRNA^∗^ have shown that the abundance of miRNA^∗^s and their biological functionality are not an occasional event but are universal in plant species. Based on the discoveries to date regarding the characteristics of miRNA^∗^ accumulation in specific tissues, immune responses to biotic and abiotic stresses, regulation of growth and development and co-regulation with mature miRNA, the miRNA^∗^ mediating mechanism represents a complex system of gene expression regulation. Although the binding of both miRNAs and miRNA^∗^s to AGO proteins has a 5′-terminal nucleotide preference, a few miRNAs and miRNA^∗^s still do not conform to this general rule. In addition to the 5′-terminal nucleotides, many other factors may also affect RNA binding to different AGO proteins, such as secondary structure and nucleotide sequence length. Furthermore, several miRNA^∗^s, such as ath-miR393b^∗^, osa-miR810a^∗^, and osa-miR1433^∗^, are expressed in various tissues and organs but not just in special tissue, indicating their widespread activities in plants (Shao et al., 2013). While some areas remain to be explored, the exploration of miRNA^∗^ characterization and function to date has further enriched our knowledge of the complex regulatory networks of microRNA systems and provided important clues for research into organism gene expression and regulation.

## Author Contributions

W-wL wrote the manuscript. JC, Y-sL, and JM contributed in revising manuscript.

## Conflict of Interest Statement

The authors declare that the research was conducted in the absence of any commercial or financial relationships that could be construed as a potential conflict of interest.
